# Inappropriate activity of local renin-angiotensin-aldosterone system during high salt intake: impact on the cardio-renal axis

**DOI:** 10.1590/2175-8239-JBN-3661

**Published:** 2018-06-18

**Authors:** Sabrina Ribeiro Gonsalez, Fernanda Magalhães Ferrão, Alessandro Miranda de Souza, Jennifer Lowe, Lucienne da Silva Lara Morcillo

**Affiliations:** 1 Universidade Federal do Rio de Janeiro, Instituto de Ciências Biomédicas, Rio de Janeiro, RJ, Brasil.; 2 Universidade do Estado do Rio de Janeiro, Instituto de Biologia Roberto Alcântara Gomes, Rio de Janeiro, RJ, Brasil.; 3 Universidade Federal do Rio de Janeiro, Instituto de Biofísica Carlos Chagas Filho, Rio de Janeiro, Brasil.

**Keywords:** Renin, Angiotensin II, Sodium, Dietary, Kidney, Heart, Renina, Angiotensina II, Sódio na Dieta, Rim, Coração

## Abstract

Although there is a general agreement on the recommendation for reduced salt intake as a public health issue, the mechanism by which high salt intake triggers pathological effects on the cardio-renal axis is not completely understood. Emerging evidence indicates that the renin-angiotensin-aldosterone system (RAAS) is the main target of high Na^+^ intake. An inappropriate activation of tissue RAAS may lead to hypertension and organ damage. We reviewed the impact of high salt intake on the RAAS on the cardio-renal axis highlighting the molecular pathways that leads to injury effects. We also provide an assessment of recent observational studies related to the consequences of non-osmotically active Na^+^ accumulation, breaking the paradigm that high salt intake necessarily increases plasma Na^+^ concentration promoting water retention

## RESUMO

Apesar de haver uma concordância geral sobre a necessidade de redução na ingestão de sal como questão de saúde publica, o mecanismo pelo qual a alta ingesta de sal deflagra efeitos patológicos sobre o eixo cardiorrenal não está ainda completamente elucidado. Cada vez mais evidencias indicam que o sistema renina-angiotensina-aldosterona (SRAA) seja o principal alvo da alta ingesta de Na^+^. Uma ativação inadequada do SRAA tecidual pode causar hipertensão e dano ao órgão. Nós revisamos o impacto da dieta com alto teor de sódio sobre o eixo cardiorrenal, destacando as vias moleculares que causam a lesão. Também fizemos uma avaliação de recentes estudos observacionais relacionados às consequências do acúmulo de Na^+^ não osmoticamente ativo, quebrando assim o paradigma de que a alta ingestão de sódio necessariamente aumenta a concentração sérica de Na^+^, assim promovendo a retenção de água.

## INTRODUCTION

The renin-angiotensin-aldosterone system (RAAS) regulates essential functions in the organism, such as the maintenance of arterial blood pressure, Na^+^, and water balance.^1^^,^ 2 The systemic RAAS is activated when renin secretion in the juxtaglomerular apparatus of the kidney is stimulated by (1) renal artery hypotension, (2) decrease in the Na^+^ load delivery to the distal tubule that is sensed by the macula densa, and (3) activation of the sympathetic nervous system activity in response to decreased arterial blood pressure.^3^^-^ 5 In the classic view of the RAAS, renin cleaves angiotensinogen (AGT) produced by the liver, generating angiotensin I (Ang I).^6^ Angiotensin II (Ang II), which is generated via Ang I cleavage by angiotensin converting enzyme (ACE),^7^^,^ 8 acts via two main receptors: angiotensin receptor type 1 (AT_1_R), which induces vasoconstriction, anti-natriuresis, anti-diuresis, vasopressin and aldosterone release, fibrosis and cellular proliferation, while angiotensin receptor type 2 (AT_2_R), which counterbalances these effects.^9^^,^ 10 Different Ang II-derived peptides, enzymes, receptors, and routes for Ang II degradation are emerging, supporting the view of different forms of regulation within the system itself.^11^ The cardio-renal axis is of particular interest since it contains all components of the RAAS (tissue or local RAAS), especially the main counterbalance route: angiotensin converting enzyme 2, angiotensin-(1-7), Ang-(1-7) MAS receptor (ACE2/Ang-(1-7)/MAS), which are involved in organ protection.^12^^-^ 15

There is a growing evidence that tissue RAAS behaves oppositely to renin plasma levels during a high salt diet (HSD).^16^^,^ 17 It has been hypothesized that this inappropriate activation of tissue RAAS is related to the pathology of cardio-renal diseases.^18^^,^ 19 The aim of this review was to investigate the association of a HSD and local RAAS with cardiac and renal disease. To provide up-to-date information, 79 relevant English language publications were selected from MEDLINE/PubMed from January 1, 1995 to July 28, 2016, using the key words: renin, angiotensin II, angiotensin-(1-7), high salt diet, kidney, and heart.

## DISCUSSION

### HIGH SALT INTAKE AND THE IMPACT ON RAAS

In general, Na^+^ intake is on average far above the 1.5-2 g/d dose recommended by the American Heart Association and the World Health Organization. Most countries consume more than double the value.^20^^,^ 21 The systemic RAAS is profoundly influenced by dietary salt intake. Under normotensive conditions, HSD inhibits the systemic RAAS while low salt diet (LSD) activates this system.^16^^,^ 22 Decreased body Na^+^ content directly influence the extracellular volume impacting renal sympathetic activity, pre-glomerular vascular baroreceptors, and the macula densa cells, and finally renin is released by the juxtaglomerular cells of the afferent arterioles.^3^^-^ 5 However, tissue RAAS components are overexpressed in salt-sensitive animal models of hypertension or in salt-sensitive hypertensive patients^17^^,^ 23 suggesting the involvement of different molecular mechanisms, which are not completely understood. The ablation of renin in renal collecting ducts of mice in an Ang II infusion hypertensive model attenuates blood pressure and renal damage.^24^ However, in a DOCA salt hypertension model, collecting duct renin is not essential to the development of hypertension and renal injury.^25^

The end point for the impairment of RAAS is AT_1_R activation. In Ang II-dependent malignant hypertension in Cyp1a1-Ren2 transgenic rats^26^, it was demonstrated that HSD, along with chronic administration of the AT_1_R antagonist, attenuates the increased systolic blood pressure and intra-renal Ang II levels, demonstrating the importance of AT_1_R in the local RAAS effect. Table 1 presents a summary of salt-sensitive hypertension rat models and the inappropriate tissue RAAS activation leading to impaired Na^+^ excretion and development of hypertension.

**Table 1:** Overview of the HSD effects in different rat models.

Rat model of HS intake	Type/Treatment	RAAS response	Cardiovascular and/or kidney responses	Ref.
Cyp1a1-Ren2 transgenic rats	HSD intake	Increased intra-renal Ang II levels	Augmented SBP	26
Wistar rats	Intra-renal Ang-(1-7) infusion plus HSD	Depressed plasma renin and Ang-(1-7) levels	Attenuated diuresis and natriuresis in comparison to LSD	27
	Left uninephrectomized subjected to HSD	Increased glomerular ACE/ACE2 ratio	Glomerulosclerosis, kidney hypertrophy and renal oxidative stress	30
Zucker rats	Lean rats subjected to HSD	Increased renal ACE and AT1BR, and decreased renin	Lean and Obese rats: augmented MAP	28
	Obese rats subjected to HSD	Increased renal ACE and Ang II in contrast to decreased ACE2, AT2R, and MAS		
SHR rats	HSD intake	Decreased ACE2 expression	Glomerular hypertrophy, loss of morphological integrity of the podocyte and augmented proteinuria.	31
	HSD intake	Increased plasma renin concentration and decreased MAS receptor expression	Increased blood pressure and renal nitroxidative stress, proteinuria, and decreased renal blood flow.	32
Sprague-Dawley rats	Ang II infused rats subjected to HSD	Exacerbated urinary AGT excretion	Exacerbated SBP, proteinuria, greater collagen deposition, mesangial expansion, interstitial cell proliferation, and macrophage infiltration.	33
	Uninephrectomized rats subjected to HSD	Decreased plasma aldosterone levels	Increased SBP, proteinuria, glomerular and interstitial injury and macrophage infiltration in kidney.	35
Dahl rats	Dahl-RS rats subjected to HSD	Suppressed plasma aldosterone.	Increased SBP	34
	Dahl-SS rats subjected to HSD	Increased plasma aldosterone levels, (pro)renin, (pro)renin receptor, angiotensinogen, ACE, AT1R and AT2R in adrenal glands.	Increased SBP and promotes left ventricular systolic dysfunction.	

HSD: high salt diet; HS: high salt; SBP: systolic blood pressure; LSD: low salt diet; MAP: mean arterial pressure; SHR: spontaneously hypertensive rats; AGT: angiotensinogen; Dahl-RS: Dahl rat hypertensive rat salt-resistant; Dahl-SS: Dahl rat hypertensive rat salt-sensitive.

To make this scenario worse, the tissue ACE/Ang II/AT_1_R counteracting route ACE2/Ang-(1-7)/MAS seems to be suppressed by HSD, which in turn could be related to augmented blood pressure. O'Neil *et al*.^27^ proposed that the renal hemodynamic and excretory responses to locally administered Ang-(1-7) is dependent on the level of Na^+^ intake and indirectly on the degree of activation of the tissue RAAS. The authors elegantly demonstrated that during a HSD, Ang-(1-7) had no effect on glomerular filtration rate, whereas the diuresis and natriuresis were attenuated compared with those in rats fed either a normal diet or LSD. This effect was independent of increases in mean arterial pressure and plasma renin. Indeed, Ang-(1-7) were highest in rats on LSD and depressed in rats on HSD^27^. In lean Zucker rats receiving 8% HSD for 2 weeks, renin and Ang-(1-7) levels were decreased in kidney cortex, while Ang II levels were the same as the control group.^28^ It was also demonstrated that Ang-(1-7) acts as a negative modulator of aldosterone secretion, since short-term LSD enhanced both plasma renin activity and blood pressure. However, this response was completely preserved during concomitant continuous Ang-(1-7) infusion, whereas the increase in aldosterone was markedly attenuated.^29^

Altogether, it is possible to postulate that the ratio ACE/ACE2 is the cornerstone for Na^+^-mediated actions. Indeed, it was shown that in male Wistar rats fed with control diet (0.2% NaCl), HSD (1.2% NaCl) and a very HSD (8.2% NaCl), ACE2 reduction is dependent on Na^+^ intake, leading to a proportional increase in the glomerular ACE/ACE2 ratio, inducing glomerular oxidative stress via Ang II.^30^ In male spontaneously hypertensive rats (SHR) under normal salt (0.3%), low salt (0.03%), or HSD (3%), it was observed that HSD induced glomerular hypertrophy and proteinuria, with a decrease in ACE2 expression, whereas LSD attenuated renal dysfunction and proteinuria due to a decrease in ACE/ACE2 protein and activity ratio within the kidney mediated by increased cubilin expression.^31^

This hypothesis was confirmed by the administration of a HSD to SHR animals^32^, which exacerbated hypertension and promoted a decrease of renal blood flow, and an increase in proteinuria and renal nitro-oxidative stress. Those events were related to the suppression of the ACE2/Ang-(1-7)/MAS axis. There was no change in plasma Ang II nor renal AT_1_R expression.^32^ Without the protective arm of the RAAS, the net result is catastrophic as demonstrated in the salt-sensitive, Ang II-dependent hypertension model: the development of malignant hypertension associated to kidney damage.^33^ Inappropriate RAAS activation was related to increased urinary angiotensinogen and intra-renal Ang II.^33^

The question that has emerged is how aldosterone is regulated since HSD leads to an inappropriate activation of ACE/Ang II/AT_1_R. The time course of changes in adrenal aldosterone biosynthesis under HSD conditions was evaluated by Morizane *et al.* 34 The time course was compared using the salt-sensitive and salt-resistant Dahl rat strains (Dahl-SS and Dahl-RS rat, respectively). Dahl-RS rats maintained suppression of aldosterone biosynthesis during HSD. In contrast, Dahl-SS rats presented a delayed and paradoxical increase in aldosterone biosynthesis after HSD intake. The authors attributed this late response to an upregulation of local RAAS components (ACE/Ang II/AT_1_R).

**Figure 1:**
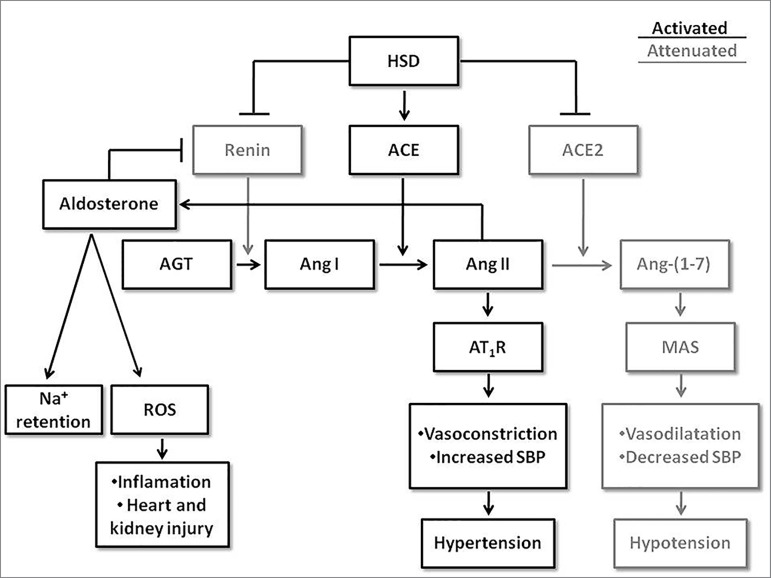
Intra-organ activation course of the two main arms of the renin-angiotensin-aldosterone system in HSD: ACE/Ang II/AT1R and ACE2/Ang-(1-7)/MAS receptor. ACE/ACE2 ratio determines which angiotensin peptide will be mainly formed: Ang II or Ang-(1-7). During HSD intake the pathway in black (ACE/Ang II/AT1R) is exacerbated due to the local increase of its components or due to decrease of ACE2/Ang-(1-7)/MAS axis (in gray). HSD: high salt diet; ACE: angiotensin converting enzyme; ACE2: angiotensin converting enzyme 2; AGT: angiotensinogen; Ang: angiotensin; AT1R: angiotensin receptor type 1; MAS: angiotensin-(1-7) receptor; SBP: systolic blood pressure; ROS: reactive oxygen species.

Indeed, Kawrazaki *et al.* 35 demonstrated that aldosterone receptor activation and HSD intake induce inflammation and oxidative stress. In young (3-week-old) and adult (10-week-old) uninephrectomized Sprague-Dawley rats fed with a HSD, the aldosterone-induced organ damage was attenuated with eplerenone (aldosterone receptor antagonist), olmesartan (AT_1_R antagonist), and FAD286 (aldosterone synthase inhibitor) treatment. It was suggested that severe hypertension and organ injury in young rats after HSD intake was primarily due to aldosterone receptor activation and secondarily to AT_1_R activation.^35^ Figure 1 summarizes the course of activation of the two main arms of RAAS: ACE/Ang II/AT_1_R and ACE2/Ang-(1-7)/MAS in tissue. We highlighted in this figure the ACE/ACE2 ratio determining the fate of the system during HSD intake and the AT_1_R responses as the end point of the inappropriate RAAS activation.

### HIGH SALT INTAKE AND THE IMPACT ON THE CARDIO-RENAL AXIS

In order to define and characterize Na^+^ sensitivity and blood pressure resistance in humans, a study was conducted with normal and hypertensive subjects and demonstrated the association among the inability to excrete Na^+^, cardiac mortality, and blood pressure.^36^

Survival curves of normotensive salt-sensitive subjects presented similar mortality index in comparison to hypertensive patients.^37^ In contrast, salt-resistant normotensive subjects presented increased survival. These observations provide evidence of a relationship between salt sensitivity and mortality that is independent of elevated blood pressure but predisposes to hypertension in the elderly. This predisposition could be related to the deleterious Na^+^ effect in the kidney throughout the years, as in the popular saying "water dropping day by day wears the hardest rock away". A study with 7850 subjects of 28-75 years of age from Netherlands demonstrated that Na^+^ intake is related to urinary albumin excretion, especially in subjects with a higher body mass index^38^. Hyper filtration was observed in normotensive Type I diabetes mellitus under LSD.^39^ A follow-up study with 47 healthy men from Naples demonstrated that the group with the highest salt sensitivity showed higher blood pressure, glomerular filtration rate, and absolute proximal sodium reabsorption during the habitual HSD compared with the least salt-sensitive group.^40^ Based on observations in humans, the World Health Organization states that the reduction of salt intake is the health strategy with the best cost-benefit ratio, preventing the development of non-communicable diseases such as hypertension, and cardiovascular and renal diseases.^41^ The global goal is to reduce daily salt intake by at least 30% per person by 2025.^42^ Therefore, the potential mechanisms altered by increased Na^+^ intake should be completely investigated.

Rat models have been used to reproduce humans observations and determine the influence of the HSD intake in the cardio-renal axis. Kidney co-transplant between Dahl-SS and -RS strains rats demonstrated the close relationship of the triad HSD intake, kidney response, and salt-sensitive hypertension. When exposed to a HSD, the Dahl-SS rat developed hypertension and reduced Na^+^excretion, while the Dahl-RS rat developed hypertension only after receiving the kidney from the Dahl-SS rat.^43^ Accordingly, Dahl-SS rats presented decreased systolic blood pressure after receiving Dahl-RS rat kidney.^44^^,^ 45

The kidney plays a major role in fluid homeostasis, controlled by tubular reabsorption of filtered solutes and water.^46^ Reabsorption of Na^+^ via transcellular pathway occurs via Na^+^ extrusion by basolateral (Na^+^+K^+^)-ATPase and Na^+^-ATPase, which allows passive apical entry via channels or exchangers.^46^^-^ 48 Na^+^/Ca^2+^ exchanger is a plasma membrane transporter that pumps Ca^2+^ out of the cell and Na^+^ into the cell, under physiological conditions.^49^ Thus, the chronic salt loading in rats leads to an increase of Na^+^ filtration and reabsorption due to an increased activity of the renal (Na^+^+K^+^)-ATPase^50^ and intracellular Ca^2+^ overload through the reverse mode of the Na^+^/Ca^2+^ exchanger.^51^ The result of Ca^2+^ overload in kidney epithelial cells is related to apoptosis and necrosis, augmented oxidative stress, and fibrosis, leading to a reduced kidney function.^52^ It is worth mentioning that both (Na^+^+K^+^)-ATPase activity and intracellular Ca^2+^ homeostasis are targets of AT_1_R activation.^11^^,^ 46 Indeed, in the salt-sensitive Ang II-dependent hypertension, kidney injury, and exacerbation of hypertension were attributed to elevated levels of intra-renal Ang II, augmented urinary angiotensinogen, and macrophage infiltration in the interstitial area.^33^

The gradual and silent reduction in kidney function leads to a proportional increase in extracellular volume, which in turn impacts the cardiac workload. It was demonstrated that a HSD intake might increase the risk of cardiovascular diseases and stroke. A reduction of 5 g a day in salt intake is associated with a 23% decrease in the rate of stroke and 17% decrease in the rate of cardiovascular disease.^53^ Hemodynamic abnormalities as a result of cardiac overwork result in sympathetic activation and RAAS activation. Initially, both mechanisms act as an acute compensatory response, but prolonged activation contributes to the progression of heart failure.^54^ Indeed, cardiac hypertrophy induced by HSD is associated to augmented cardiac RAAS in different rat models and humans.^55^^-^ 58

Irrespective of the origin of RAAS components (hormonal or local), the majority of Ang II in the heart is produced *in situ*, especially in pathological conditions such as myocardial infarction and heart failure^59^^-^ 61 due to an augmented ACE expression and activity.^62^ The elevated cardiac levels of Ang II and aldosterone (compared to plasma levels) observed in the hearts of the Dahl-SS rats were related to the severity of vascular maladaptations and to the maintenance of hypertension.^56^ Ang II and aldosterone accumulation in the heart leads to TGF-β overexpression, increase in cardiac protein, and fibrosis. 63 ^,^ 64

Elevated plasma Na^+^ concentrations have been shown to stiffen vascular endothelial cells (EC) accompanied by a decrease in the bioavailability of nitric oxide (NO).^65^ This mechanical alteration is associated to the dysfunction of the EC since physiologically NO is released by shear stress causing vasodilation.^66^ In addition, the Na^+^ channel present in the vascular endothelium (EnNaC) seems to be the crucial mediator of the endothelial salt sensitivity, leading to vascular stiffening and endothelium nitric oxide synthase phosphorylation (eNOS), decreasing NO production.^66^ Spironolactone (aldosterone receptor antagonist) and amiloride (EnNaC blocker) lowered EnNaC abundance and prevented endothelial stiffening.^67^

### BREAKING THE PARADIGM

Alterations of Na^+^ homeostasis can cause water retention in the intravascular compartment increasing systolic blood pressure, as occurs in Na^+^-sensitive hypertension.^68^ As proposed by Arthur Guyton in the 1960's, this observation was related to abnormal pressure-natriuresis curves in various forms of hypertension.^69^ However, the inability to excrete Na^+^ does not necessarily infers in an increase in plasma volume, a phenomenon named the "Lag Phenomenon".^70^ In this condition, blood pressure is established at a new higher level due to an enhanced peripheral vascular resistance and no alteration in plasma volume and Na^+^ balance.^71^^,^ 72 Indeed, extracellular Na^+^ concentration did not change in similar conditions in another study.^73^ Therefore, the theory of the third compartment that proposed that Na^+^ is locally stocked seems to be retrieved. Dahl-SS rat in a HSD intake presented a reduced ability to excrete Na^+^ leading to Na^+^ and water excess that was characterized by bone, cartilage and mixed connective tissue storage.^74^ Tissue Na^+^ accumulation was detected in skeletal muscle^68^ and in the skin trapped in the negative charges of glycosaminoglycans (GAG).^74^^,^ 75 This osmotically inactive storage could function as a buffer that receives Na^+^ from an overloaded extracellular space. Titze *et al.* proposed that in salt-sensitive hypertension, there is a dysfunction in GAG, releasing osmotically active Na^+^ and promoting organ damage.^74^ A clinical study using magnetic resonance imaging (Na-MRI) showed that men have a higher capacity to store non-osmotically Na^+^ than women, which was also observed in patients under dialysis. The increase was age-dependent and higher in hypertensive than normotensive subjects.^76^ The patho-physiological function of interstitial Na^+^ storage is still not well characterized. Luft, in his editorial commentary^75^, reported that locally increased Na^+^ concentration leads to disrupted glycocalyx accompanied by a decreased NO production and EnNaC activation leading to endothelium stiffness as referred above. Understanding the molecular mechanism involved in the non-osmotically Na^+^ stores could be another point of pharmacological interventions.

It has been demonstrated that Na^+^ *per se* modulates the pharmacology efficacy of RAAS blockers. In an animal model of adriamycin-induced nephropathy, low Na^+^ potentiates the renal protective effect of RAAS-blockade by decreasing proteinuria, blood pressure, and glomerulosclerosis.^77^ In humans, Na^+^ restriction produces a potential antiproteinuric effect leading to long-term cardiovascular and renal protection.^78^ This observation was presented in a clinical cohort study with Na^+^ intake in chronic kidney disease patients and in renal transplant populations. By 24-h urinary collections, a direct association between proteinuria and Na^+^ intake was found. In addition, systolic blood pressure was usually Na^+^-sensitive.^79^ Renal or cardiovascular complications increased by approximately two times during HSD intake in comparison to LSD intake in patients treated with AT_1_R blocker.^77^

## CONCLUSION

Information on the impact of salt intake on the course of heart and kidney disease is still unclear but it indicates that the cornerstone for tissue inappropriate activation of RAAS is the ACE/ACE2 ratio, leading to augmented local Ang II and AT_1_R activation. For this reason, a reduction in salt consumption could enhance the effectiveness of the therapeutic arsenal targeting the RAAS. This review showed that local RAAS responds differently to salt than the systemic system. Even though the systemic ACE/Ang II/AT_1_R pathway is pharmacologically attenuated, HSD favors ACE over ACE2 in the tissue, especially in the cardio-renal axis. Unbalanced ACE/Ang II/AT_1_R over ACE2/Ang-(1-7)/MAS locally could be related to the progression of heart and kidney failure.

### LIST OF ABBREVIATIONS

Classic or systemic RAAS: renin-angiotensin-aldosterone system activated by renin release from juxtaglomerular apparatus, acting on circulating angiotensinogen.

Tissue or local RAAS: renin-angiotensin-aldosterone system activated by tissue renin, acting on locally produced angiotensinogen.

ACE/Ang II/AT_1_R axis: vasoconstrictor, anti-natriuretic, and anti-diuretic arm of the RAAS related to organ injury.

ACE2/Ang-(1-7)/MAS axis: vasodilator, natriuretic, and diuretic arm of the RAAS, related to organ protection.
